# Asynchronous Telemedicine: A Systematic Literature Review

**DOI:** 10.1089/tmr.2023.0052

**Published:** 2023-12-21

**Authors:** Nathan Culmer, Todd Brenton Smith, Catanya Stager, Andrea Wright, Abigail Fickel, Jet Tan, Carlos (Trey) Clark, Hannah Meyer, Katherine Grimm

**Affiliations:** ^1^ College of Community Health Sciences, The University of Alabama, Tuscaloosa, Alabama, USA.; ^2^ Capstone College of Nursing, The University of Alabama, Tuscaloosa, Alabama, USA.; ^3^ The University of Alabama, Tuscaloosa, Alabama, USA.

**Keywords:** systematic review, asynchronous telemedicine, store-and-forward, e-visit

## Abstract

**Background:**

Asynchronous telemedicine (ATM), which describes telemedical interaction between a patient and provider where neither party communicates simultaneously, is an important telemedicine modality that is seeing increased use. In this article, we summarize the published peer-reviewed literature specifically related to ATM to (1) identify terms or phrases that are used to describe ATM, (2) ascertain how this research has thus far addressed the various aspects of the quadruple aim of medicine, and (3) assess the methodological rigor of research on ATM. We also divided the literature into pre- and post-COVID-19 onset periods to identify potential variations in the literature between these two periods.

**Methods:**

This systematic literature review follows the Preferred Reporting Items for Systematic Reviews and Meta-Analyses guidelines. The literature search, utilizing multiple databases and applying inclusion and exclusion criteria, initially produced 2624 abstracts for review. De-duplication and screening ultimately yielded 104 articles for data extraction.

**Results:**

“Store-and-forward” and variations of “e-visit” were the most frequently used alternative terms for ATM. Care quality was the most frequently addressed aspect of the Quadruple Aim of Medicine—more than double any other category—followed by patient satisfaction. We separated cost of care into two categories: patients' cost of care and providers' cost to provide care. Patient cost of care was the third most addressed aspect of the Quadruple Aim of Medicine followed by provider well-being and provider's cost to provide care. Methodological rigor of the studies was also addressed, with only 2 quantitative studies ranked “Strong,” 5 ranked “Moderate,” and 97 ranked “Weak.” Qualitative studies were generally acceptable but struggled methodologically with accounting for all participants and articulation of results.

**Conclusions:**

Although “store-and-forward” is somewhat more frequently used in the studies included in this review, variants of “e-visit,” are growing in recent usage. Given the relative newness of modality, it is not surprising that quality of care is the most researched aspect of the Quadruple Aim of Medicine in ATM research. We anticipate more balance between these areas as research in this field matures. Primary areas of research need currently relate to practitioners—specifically their costs of providing care and well-being. Finally, future ATM research needs to address research challenges of selection bias and blinding in quantitative studies and improved participant tracking and articulation of both study design and results in qualitative studies.

## Background

Telemedical solutions (e.g., telemedicine) continue to transform health care services provided by medical providers, especially considering the recent international COVID-19 pandemic and the call for innovative health care delivery methods.^1–3^ The widespread use and acceptance of telemedicine have given rise to virtual care procedures^4^ and integration of telemedicine within established health systems.^5^ However, this rapidly accelerating implementation of telemedicine has resulted in changes arriving quicker than studies can be conducted,^6^ especially those considering the Quadruple Aim for Health care, which promotes care quality and satisfaction while reducing the cost of care and improving the well-being of providers.^7^

As the primary purpose of telemedicine is to deliver remote health care services via telecommunication technology,^8^ its implementation is directed chiefly at rural populations and patients with conditions that make access to health care services, such as specialists, difficult.^9^ Telemedicine also offers lower average costs and increased convenience for patient-provider visits, thus making it an increasingly popular alternative.^10^ In addition, with telemedicine, certain providers (e.g., specialists) can also utilize the telemedicine technologies to provide medical insights and information to other medical providers who may not be specialists or who may be located in a rural area.^11^

More recently, the COVID-19 pandemic has caused an even more significant increase in telemedicine demand and usage due to social distancing and quarantining.^12^ For example, the Centers for Medicare and Medicaid Services has reported unprecedented recent increases in telemedical services, with ∼13,000 users in 1 week before the pandemic and nearly 1.7 million users in the last week of April, 2020.^13^

The delivery of telemedical health care is generally divided into three distinct mechanisms: synchronous, remote patient monitoring (RPM), and asynchronous.^14^ Synchronous telemedicine occurs when the provider and patient communicate in real-time over distance using technology.^9^ On the contrary, RPM involves a provider remotely monitoring a patient's condition using automated systems, which collect and deliver specific data, tests, and/or images.^11^ Importantly, RPM does not necessarily imply a back-and-forth discussion between physician and patient. However, RPM may be followed by future health care interactions which may include in-person contact, synchronous, or asynchronous telemedicine (ATM). The third method, ATM, is generally defined as any telemedical interaction between a patient and provider where neither party is communicating simultaneously.

Specifically, Abbasi-Feinberg^15^ defines asynchronous telecommunication as “medical information [being] stored and forwarded to be reviewed by a physician or healthcare practitioner at a distant site [where] the medical information is reviewed without the patient being present.” This absence of the need for both patient and provider to be online simultaneously gives patients living in remote areas, with reduced or unreliable connectivity, the opportunity to receive affordable, flexible, and convenient health care services.

In this article, we will summarize the published peer-reviewed literature specifically related to ATM through (1) identifying terms or phrases used to describe ATM, (2) determining the different aspects of the Quadruple Aim of Health care (which has been used as a framework to evaluate studies related to ATM^16–19^) in this field, and (3) examining the methodological rigor of published research on this topic. In addition, we have divided the literature reviewed into the pre- and post-COVID onset periods to examine differences that may have occurred within this literature between these two time periods.

This systematic review is distinct among systematic reviews of ATM in that it focuses on communication between providers and patients as opposed to communication between providers and providers.^19–21^ Moreover, the focus of this review is not restricted to a particular health condition,^22^ condition type,^23^ or specialty.^24,25^ Also, several recent systematic reviews focus on certain aspects of the Quadruple Aim Framework,^26–28^ but we only found one systematic review,^19^ published in 2014, that included all four aspects of this framework. Furthermore, no other systematic review using the Quadruple Aim Framework examines ATM in terms of the distinct costs borne by providers in providing care asynchronously.

Finally, our study provides comparisons of ATM before and after the onset of the COVID-19 pandemic. We did identify one other study^27^ which conducted a pre- and post-COVID-19 onset comparison on the clinical aspects of ATM, but it had a more limited date range than this review and did not include the remaining aspects of the Quadruple Aim Framework.

## Materials and Methods

**FIG. 1.**
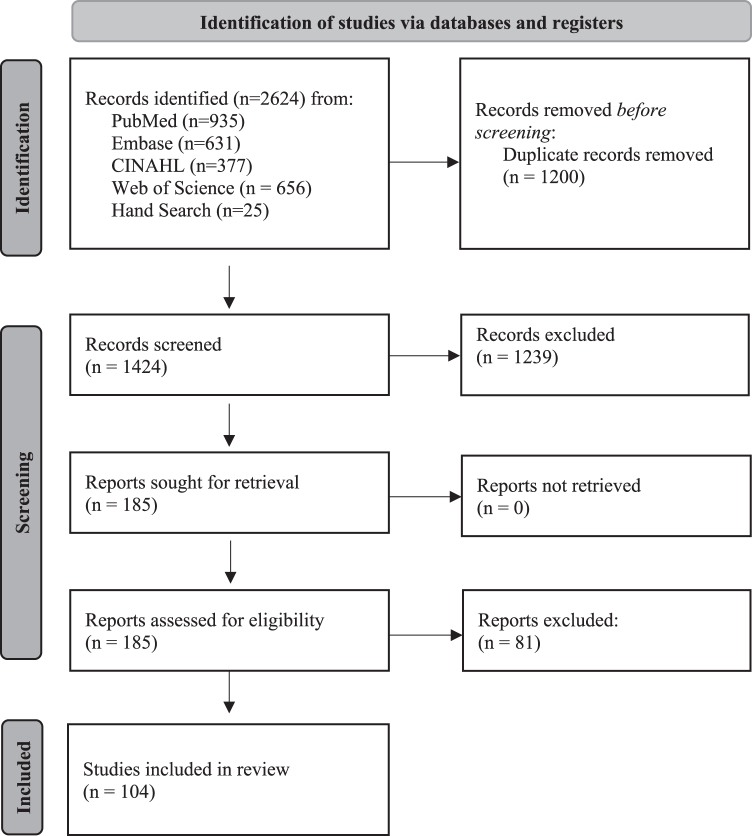
PRISMA diagram. PRISMA, Preferred Reporting Items for Systematic Reviews and Meta-Analyses.

This systematic literature review follows the Preferred Reporting Items for Systematic Reviews and Meta-Analyses guidelines (Fig. 1).^29^ We searched Medline (all PubMed), CINAHL, EMBASE, and Web of Science databases for terms related to “telemedicine” and “asynchronous” (including “store and forward”), with no date limitations, which yielded 2599 records (Table 1). An additional 25 records were found through hand-searching, resulting in a total of 2624 records. Using Rayyan abstract review software,^30^ 1200 duplicates were identified and removed from the search, leaving 1424 abstracts to review.

**Table 1.** Asynchronous Telemedical Search Terms

Search terms and subject headings (PubMed):	
Telemedical search terms	Asynchronous search terms
“telemedicine”[mesh] OR “remote consultation”[mesh] OR telemed^*^[tw] OR tele-med^*^[tw] OR telehealth^*^[tw] OR tele-health^*^[tw] OR telemonitor^*^[tw] OR tele-monitor^*^[tw] OR telecare^*^[tw] OR tele-care^*^[tw] OR telehome^*^[tw] OR tele-home^*^[tw] OR teleteach^*^[tw] OR tele-teach^*^[tw] OR teletrain^*^[tw] OR tele-train^*^[tw] OR ehealth^*^[tw] OR e-health^*^[tw] OR mhealth^*^[tw] OR m-health^*^[tw] OR mobile-health^*^[tw] OR remote-consult^*^[tw] OR electronic-consult^*^[tw] OR e-consult^*^[tw] OR econsult^*^[tw] OR distance-consult^*^[tw] OR text-messag^*^[tw] OR SMS[tw]	asynchronous^*^[tw] OR store-and-forward[tw] OR pre-record^*^[tw]

These 1424 abstracts were independently reviewed relative to our inclusion criteria by at least two members of our research team using Rayyan.^30^ Once independent reviews were completed, all conflicts between reviewers were resolved by a process of consensus, leaving 185 articles for full review and data extraction.

Included articles (1) were in English; (2) contained an abstract; (3) were published in 2000 or later; (4) described a health care encounter via technological means between physician/health care provider and patient (not provider-to-provider communication in contrast to Liddy et al.,^19^ who focused on provider-to-provider telemedicine); (5) described a health care encounter via asynchronous technological means; and (6) focused on empirical analyses of data. Articles were excluded if they were (7) a systematic review, critical review, meta-analysis, editorial, or case-study; (8) a conference proceeding; or (9) designed to test the concordance, or level of agreement, between telemedical and face-to-face (FTF) encounters, without providing actual care.

### Reporting criteria/coding

During full-text review, 81 additional articles were excluded from for not meeting inclusion criteria, leaving 104 articles in the final analysis. Subsequently, data extraction was conducted independently by at least two members of the research team, with all conflicts resolved by consensus.

Data were coded according to the following criteria: study characteristics (sample size, date of publication, location(s) of study, etc.); patient demographic characteristics; care encounter characteristics (e.g., specialties involved, nature of the provider/patient relationship, number of visits in the interaction, etc.); data related to reasons for patient use, practitioner activities, and patient outcomes; and data related to the quality and cost (to both patient and provider) of care, satisfaction with the care provided, and any impacts care had on provider well-being.

During the data extraction process, we also examined the published date for each article and divided the article into two subsets, those published before 2020 (roughly the pre-COVID-19 period) and those published after 2020 (roughly the post-COVID-19 period).

### Methodological rigor

For quantitative studies and the quantitative components of mixed-method studies, we used the Effective Public Health Practice Project (EPHPP) Quality Assessment Tool, which classifies studies based on selection bias, study design, handling of confounders, blinding, data collection methods, and withdrawals and dropouts to assess methodological rigor and generate a global rating of strong, moderate, or weak for each article.^31,32^ For qualitative studies and the qualitative components of mixed-method studies, we used the Critical Appraisal Skills Programme (CASP) Qualitative Studies Checklist,^33^ which focuses on three themes: validity of study results (Section A: Questions 1–6), the study design and results themselves (Section B: Questions 7–9), and the generalizability of results (Section C: Question 10).

With one exception, questions on this assessment tool are answered with the options of “Yes,” “Can't Tell,” or “No” with an option to comment. We modified the last question “How valuable is the research” to include the same response options as it natively only allows for comments—making any aggregation of responses on this question unruly. As the designers of the CASP do not suggest a scoring system, we reported the aggregated number of responses by question within each category to provide a gestalt view of methodological rigor.

## Results

### Study characteristics

Sample sizes of the included studies ranged widely, with 82 (78.8%) having less than 500 (or in the test group, when applicable) and 11 (10.6%) having more than 3000. Of these 11 studies, 6 included very large data sets (compared to other included studies), which precluded a meaningful summary of the overall average sample size, and 4 of these 6 did not measure asynchronous telemedical visits as their primary outcome measure. Twenty-eight (26.9%) studies had a sample of something other than unique patients. Among those referring to something other than patients as the basic unit of analysis, 14 (13.5%) used number of e-Visits without specifying unique patient numbers, 6 (5.8%) used providers as study subjects, 2 (1.9%) used ears, and 1 each (1.9%) used insurance claims, clinic sites, users (of the system), hospitalizations, parents, and parent/child dyads.

The number of publications related to ATM tended to increase over time, with 13 (12.5%) articles included in this review published between 2000 and 2009, 50 (48.1%) between 2010 and 2019, and 41 (39.4%) between 2020 and 2022. Sixty (57.7%) of the 104 included studies were grant funded, and this pattern was similar between studies published both pre- and post-January 1, 2020. Most included studies were conducted in North America (*n* = 62, 59.6%), primarily in the United States (59, 56.7%), with most remaining studies conducted in Europe (25, 24.0%).

Study duration varied from a single instance (a one-time survey) to 8 years, although most studies were a year or less (69, 66.3% of total) and 13 (12.5%) were 1–2 years in length. Seven (6.7%) did not specify a study length. Fifty-five studies (52.9%) were retrospective and 49 (47.1%) were prospective. Eighty-seven (83.7%) were quantitative, 9 (8.7%) were qualitative, and 8 (7.7%) were mixed methods.

### Participant characteristics

Fifty (48.1%) studies included adult participants, 5 (4.8%) included pediatric participants, and 24 (23.1%) reported pediatric and adult participants, while 25 (24.0%) either did not specify or were unclear, indicative of largely adult participant pools. Race was reported in only 31 (29.8%) of included studies.

In most, but not all, studies, white participants outnumbered other reported races. When reported, race involved between one and five races, not including those who were categorized as “Other” or “Not reported.” Eighty-six included studies involved both male and female participants. Five (3.6%) involved only one gender. One study (1.0%) reported more than two genders. Twelve studies (11.5%) did not report on the gender composition of their study participants. Twelve (11.5%) were conducted in urban settings, while 13 (12.5%) were conducted in rural settings. Twenty-one (20.2%) of the studies reported being conducted in both rural and urban settings and 58 (55.8%) did not specify the setting of their study. Only 15 studies (14.4%) reported on patient income and 22 (21.2%) reported on patient insurance status.

### Characteristics of care

Regarding characteristics of care, we extracted data related to alternative terms for ATM; the context of care (including health specialties and provider credentials, the nature of interaction between patients and providers, and results of care); and examined elements of the quadruple aim of medicine.

### Alternative terms

We identified 54 unique terms that were used to identify ATM. The most commonly used single term was “store-and-forward” (*n* = 37), followed by “electronic visit” (*n* = 14; Table 2). Notably, “store-and-forward” and its variations were used more frequently before the pandemic (*n* = 28 before 2020; *n* = 9 in 2020 and beyond).

**Table 2.** Terms Used to Describe Asynchronous Telemedical Encounters Between Providers and Patients

Category name	De-duplicated list of terms	Term count	Category subtotal
Asynchronous	Asynchronous	1	
	Asynchronous delivery	1	
	Asynchronous messaging	1	
	Asynchronous tele-exercise	1	
	Asynchronous teledermatology	1	
	Asynchronous telehealth	1	
	Asynchronous virtual care	1	
	Asynchronous virtual visits	1	
	Tele-rehabilitation	1	
	Subtotal		9
e-Visit	Delayed-time electronic visit	1	
	e-therapy	1	
	e-Visit	9	
	eHealth	1	
	Electronic visit	13	
	Virtual visit	2	
	Subtotal		27
Email	E-mail	1	
	Email consultation	1	
	Subtotal		2
Mobile App	Customized smartphone app platform	1	
	Mobile app	3	
	Mobile application	4	
	Subtotal		8
Portal	Patient portal messaging	1	
	Secure Patient Portal	1	
	Secure web-based portal	1	
	Secure web-based portal with message, audio, and video capabilities	1	
	Web portal	1	
	Subtotal		5
Remote monitoring	Remote monitoring	1	
	Telehealth remote monitoring	1	
	Subtotal		2
Secure messaging	Chat based patient-provider interaction	1	
	Digital communication	2	
	Secure message	1	
	Secure message exchange	2	
	Subtotal		6
Store-and-forward	Store and forward	37	
	VDOT	1	
	Subtotal		38
Teleconsulting	Asynchronous Online Consultation	1	8
	Asynchronous teleconsultation	1	
	Online Consultation	1	
	Teleconsultation	3	
	Web-based Asynchronous Teleconsulting	2	
	Subtotal		
Telehealth	Telehealth	1	2
	Teleophthalmology kiosk	1	
	Subtotal		
Text messaging	Automated Text Messaging System	1	9
	Bi-directional text messaging	1	
	Multimedia message service (text msg apps)	1	
	Text messaging teleconsultation	1	
	Text-based	5	
	Subtotal		
Web-based	Internet-based	3	18
	Online care	1	
	Secure website	1	
	Web-based	9	
	Web-based connected-health platform	3	
	Website platform	1	
	Subtotal		
Other	Computer therapy	1	3
	Direct to Patient	1	
	Time delayed	1	
	Subtotal		
Total			137
Number of categories			13

VDOT, video directly observed therapy.

Because many of these terms are similar or described similar processes, we chose to group them thematically, resulting in 13 categories. “Store-and-forward” was again the most popular category (*n* = 38), followed by “E-visit” and its variants (*n* = 27). Third most common was the “Web-based” category (*n* = 18) followed by “Asynchronous” and “Text Messaging” (*n* = 9 each). Also, “Asynchronous” and “Web-based” often appear to be used as adjectives describing an existing type of care as opposed to a unique type of care in and of itself (Table 3).

**Table 3.** Summary of Findings Including Terms Used to Describe Asynchronous Telemedicine, Health Specialties Involved, and Quadruple Aim Categories

Study	ATM alternative terms	Health specialty(ies)	Sample size	Comparison group(s) size	Quadruple aim components					Sample, if not patients
					Care quality	Patient cost of care	Provider cost of care	Patient satisfaction	Provider well being	
Abbas et al. (2008)^34^	Store and forward	Cardiology	150	N/A	✓					Consultations
Adamson et al. (2010)^35^	Electronic visit	Primary care	1159	N/A		✓	✓			
Albert et al. (2011)^36^	Electronic visit	Primary care	121	N/A	✓			✓		
Alberts et al. (2021)^37^	Mobile app	Neurology	23	N/A	✓					
Armstrong et al. (2018)^38^	Web-based connected-health platform	Dermatology	148	148	✓					
Armstrong et al. (2019)^39^	Web-based connected-health platform	Dermatology	148	148	✓					
Armstrong et al. (2015)^40^	Store and forward	Dermatology	78	78	✓			✓		
Atherton et al. (2020)^41^	Email consultation	Primary care	85	N/A	✓					
Barrera-Valencia et al. (2017)^42^	Store and forward	Psychiatry	50	49	✓	✓		✓		
Bianchi et al. (2019)^43^	Store and forward	Dermatology	6879	N/A	✓					
Bini et al. (2017)^44^	Mobile application	Rehabilitation	15	15				✓		
Bosanac et al. (2018)^45^	Store and forward	Dermatology	13	13				✓		
Careyva et al. (2021)^46^	Electronic visit	Primary care	10673	31226	✓					e-Visits
Chambers et al. (2012)^47^	Store and forward	Dermatology	28	31	✓					
Chan et al. (2003)^48^	Store and forward	Pediatrics, pulmonology	5	5	✓			✓		
Chan et al. (2007)^49^	Store and forward	Pediatrics, pulmonology	47	55	✓					
Chen et al. (2004)^50^	Store and forward	Ophthalmology	113	N/A	✓					
Choi et al. (2019)^51^	Customized smartphone app platform	Cardiology, dietetics	44	44	✓			✓		
Collins et al. (2004)^52^	Store and forward	Dermatology	80	68				✓		
Compen et al. (2017)^53^	Text-based, web-based	Psychiatry	125	N/A				✓	✓	
Constantinescu et al. (2011)^54^	Store and forward	Voice therapy	17	17	✓			✓		
Costa et al. (2021)^55^	Asynchronous, asynchronous tele-exercise, mobile app (WhatsApp)	Rehabilitation	40	N/A	✓					
Costello et al. (2019)^56^	Store and forward	Dermatology	37	N/A					✓	
Courneya et al. (2013)^57^	Online care	Primary care	4008	175678	✓		✓	✓		Patient insurance claims
Dahne et al. (2021)^58^	Electronic visit, e-visit	Primary care	34	17	✓	✓		✓		e-Visits
Das et al. (2022)^59^	Web-based	Dermatology	141	22, 41^a^	✓				✓	Asynchronous visits
Datta et al. (2015)^60^	Store and forward	Dermatology	195	196	✓	✓	✓			
de Mello-Sampayo (2019)^61^	Store and forward	Dermatology	70	53		✓				
de Souza-Junior et al. (2017)^62^	E-mail	Urology	15	N/A	✓					
Dixon et al. (2014)^63^	eVisit, website platform	Primary care	175	N/A				✓	✓	
Ebert et al. (2013)^64^	Internet-based, web-based	Psychopathology	131	146	✓					
Edison et al. (2008)^65^	Store and forward	Dermatology	110	N/A	✓				✓	
Eldh et al. (2020)^66^	Digital communication	Primary care	21	N/A					✓	Health staff
Ekström et al. (2019)^67^	Time delayed, internet-based	Psychiatry, psychology	12	N/A	✓				✓	Therapists
Entezarjou et al. (2020)^68^	Digital communication, chat-based patient-provider interaction	Primary care	19	N/A	✓				✓	General practitioners and nurses
Entezarjou et al. (2021)^69^	Electronic visit; eVisits	Primary care	3847	759	✓					e-Visits
Ferrandiz et al. (2007)^70^	Store and forward	Dermatology	134	N/A	✓					
Ferrándiz et al. (2012)^71^	Store and forward	Dermatology	67	134	✓					
Fiks et al. (2018)^72^	Store and forward	Dermatology	135	N/A				✓		
Furness et al. (2021)^73^	Asynchronous delivery, mobile app, eHealth	Nutrition	19	N/A	✓					General practitioners and nurses
Garfein et al. (2020)^74^	VDOT	Pulmonology	149	N/A	✓			✓		
Godfrey et al. (2021)^75^	Direct to patient, online consultation, asynchronous online consultation	Primary care	534	N/A		✓				
Govender et al. (2018)^76^	Store and forward	Otology	146	N/A	✓					Ears
Greenwald et al. (2020)^77^	Store and forward	Dermatology	1389	N/A	✓					
Greenwood et al. (2015)^78^	Telehealth remote monitoring	Primary care	40	41	✓			✓		
Harrison et al. (2020)^79^	tele-rehabilitation, computer therapy	Speech language pathology	85	N/A	✓	✓				
Hatch et al. (2020)^80^	Store and forward, asynchronous telehealth	Neurology	575^b^	1146, 117^b^	✓					Person encounters
Hawes et al. (2018)^81^	SaF, evisit	Pharmacy	36	N/A	✓	✓	✓	✓		
Hefner et al. (2019)^82^	Patient portal messaging	Family medicine	17	N/A	✓			✓		
Herce et al. (2011)^83^	Store and forward	Pre-oral surgery	97	2550	✓			✓		
Hertzog et al. (2019)^84^	Electronic visit	Primary care	490	2201	✓					
Heyworth et al. (2014)^85^	Secure message exchange	Pharmacy	51	N/A	✓					
Hull et al. (2017)^86^	Text-based, web-based	Psychotherapy	57	N/A	✓	✓		✓		
Jannati et al. (2021)^87^	Store and forward, text messaging teleconsultation, teleconsultation	Primary care	396	N/A				✓		
Kapoor et al. (2020)^88^	Store and forward, teleophthamology kiosk	Optometry, ophthalmology	326	N/A		✓				
Kazi et al. (2021)^89^	Store and forward, asynchronous teledermatology	Dermatology	951	1672	✓	✓			✓	Asynchronous visits
Kelley et al. (2020)^90^	Virtual visit, asynchronous messaging	Primary care	912	N/A	✓	✓		✓		
Krupinski et al. (2004)^91^	Store and forward	Dermatology	50	50	✓					
Langkamp et al. (2015)^92^	Store and forward	Primary care	73	N/A	✓	✓		✓		Parents of developmentally disabled children
Lee et al. (2021)^93^	Store and forward, asynchronous virtual care, asynchronous virtual visits	Dermatology	12696	82270	✓					Asynchronous visits
Levine et al. (2018)^94^	Virtual visit	Primary care	893	893	✓					
Loane et al. (2000)^95^	Store and forward	Dermatology	96	102	✓	✓	✓			
Malgaroli et al. (2020)^96^	Multimedia message service (text message apps)	Psychiatry, psychology	475	N/A	✓					
Maloney (2019)^97^	Store and forward	Dermatology	49	N/A	✓	✓				
Maruthurkkara et al. (2022)^98^	Remote monitoring	Audiology	32^c^	N/A	✓	✓		✓		
Mirzaei et al. (2020)^99^	Text-based	Primary care	345	N/A	✓			✓		
Mollard et al. (2018)^100^	Mobile application, web portal	Rheumatology	21	15	✓					
Mostafa et al. (2020)^101^	Store-and-forward, telehealth	Dermatology	62	N/A	✓			✓		
Murray et al. (2019)^102^	Electronic visit/secure patient portal	Urology	150	300	✓					
Naeemabadi et al. (2020)^103^	Web-based connected-health platform	Rehabilitation	12	N/A	✓			✓		
Nahm et al. (2020)^104^	Store and forward	Dermatology	13	N/A	✓					
Nichols et al. (2020)^105^	Mobile application (Smartphone Asthma Management System)	Pediatrics	19	N/A				✓		Parent child dyads^d^
Obr et al. (2020)^106^	Bi-directional text messaging	Emergency medicine	390	399	✓			✓		
Parsi et al. (2012)^107^	Store and forward	Dermatology	32	32	✓	✓	✓			
Pathipati et al. (2016)^108^	Electronic visit	Dermatology	38	N/A		✓		✓		
Penza et al. (2018)^109^	e-Visit	Primary care	1009	N/A	✓	✓	✓			e-Visits
Penza et al. (2020)^110^	Electronic visit	Pediatrics	101	202, 202	✓					
Penza et al. (2021)^111^	Electronic visit	Primary care	150	150, 150	✓					
Peracca et al. (2021)^112^	Store and forward	Dermatology	44438	N/A	✓					
Player et al. (2018)^113^	e-Visit	Primary care	1565	N/A	✓	✓		✓		e-Visits
Postel et al. (2011)^114^	e-therapy	Psychiatry	1603	2990	✓					
Rajda et al. (2018)^115^	Store and forward	Dermatology	152	152		✓	✓	✓		
Ratanjee-Vanmali et al. (2020)^116^	Mobile application (WhatsApp)	Otolaryngology	31	N/A	✓			✓		
Reed et al. (2020)^117^	Secure message exchange, secure message	Endocrinology	150179	91331	✓					Hospitalizations
Rimner et al. (2010)^118^	Store and forward	Dermatology	46	N/A	✓					
Rohrer et al. (2010)^119^	e-visits (via a secure patient portal)	Primary care	390	376		✓				
Sadasivam et al. (2020)^120^	Secure web-based portal	Tobacco treatment specialists	245	N/A	✓					
Seguí et al. (2020)^121^	Teleconsultation	Primary care	18	N/A	✓					General practitioners
Seguí et al. (2020)^122^	Teleconsultation, asynchronous teleconsultation	Primary care	5382	N/A	✓					Online consultations
Shigaki et al. (2013)^123^	Secure website	Rheumatology	43	45	✓					
Solans et al. (2021)^124^	Teleconsultation	Primary care	187435	11493173	✓					eConsulta users
Stamenova et al. (2020)^125^	Electronic visit	Primary care	6355	N/A				✓		
Stamenova et al. (2020)^126^	Secure web-based portal with message, audio, and video capabilities	Pulmonology	41	81	✓					
ter Huurne et al. (2013)^127^	Web-based	Psychopathology	89	N/A	✓			✓		
ter Huurne et al. (2015)^128^	Web-based	Psychopathology	97	104	✓					
Umefjord et al. (2004)^129^	Text-based, web-based	Primary care	21	N/A					✓	General practitioners
Umefjord et al. (2008)^130^	Text-based, web-based	Primary care	38217	N/A					✓	Inquiries
Valenzuela et al. (2007)^131^	Web-based asynchronous teleconsulting	Gynecology, dermatology, gastroenterology	270	N/A				✓		Teleconsultations
Valenzuela et al. (2010)^132^	Web-based asynchronous teleconsulting	Gynecology, dermatology, gastroenterology	1624	N/A				✓		Consultations
Van Den Berg et al. (2006)^133^	Internet-based	Rheumatology	77	75	✓					
Watson et al. (2010)^134^	Electronic visit, e-Visit	Dermatology	54	67	✓			✓	✓	
Yakovchenko et al. (2019)^135^	Automated text messaging system	HCV treatment	15	15	✓	✓	✓	✓		Clinics
Yellowlees et al. (2021)^136^	Delayed-time electronic visit	Psychiatry	80	80	✓					
Yokose et al. (2020)^137^	Electronic visit	Rheumatology	62	62	✓					
Totals		32			82	23	9	39	12	
Percent of studies reporting this component of the Quadruple Aim of Medicine					78.8%	22.1%	8.7%	37.5%	11.5%	

^a^ Twenty-two visits were synchronous only, 41 were asynchronous visits that converted to synchronous.

^b^ Numbers reported reflect primary telemedicine codes reported only. Secondary codes were reported in terms of synchronous (*n* = 2433) and asynchronous (*n* = 1238) encounters but are not included in this table for clarity and parsimony.

^c^ Thirty-two participants, 52 ears.

^d^ Thirty-eight total participants, but study reported dyads (*n* = 19) as the unit of measure.

ATM, asynchronous telemedicine; HCV, hepatitis C virus.

### Context of care

#### Specialties and credentials

All but one^135^ of the 104 included articles reported providers' health care specialty, with dermatology (*n* = 28, 26.9%) and primary care (*n* = 28, 26.9%), followed by psychiatry (*n* = 6, 5.8%), as the most frequently mentioned. No other specialty was mentioned more than four times. Seventy-seven (74.0%) studies specified a physician as the primary care provider. Other providers included an assortment of nurses, nurse practitioners, pharmacists, audiologists, dieticians, therapists and other health care providers. In 10 (9.6%) studies, the credentials of the provider(s) were not specified. Thirteen (12.5%) studies involved interprofessional care teams using eight different care team configurations.

#### Patient-provider relationships

In addition, we categorized the patient-provider relationship as either episodic (intermittent or single visits) or ongoing (repeated visits over time), with more studies reporting episodic rather than ongoing overall (*n* = 56, 53.8% vs. *n* = 48, 46.2%, respectively). However, when compared between pre-COVID-19 onset and post-COVID-19 onset, the proportion of studies reporting episodic relationships decreases (*n* = 37/63, 58.7% pre-COVID-19 vs. *n* = 19/41, 46.3% post-COVID-19 onset), while the proportion of studies reporting ongoing relationships increases (*n* = 26/63, 41.3% pre-COVID-19 vs. *n* = 22/41, 53.7% post-COVID-19 onset).

### Results of care

The results of care within each study were categorized by the patients' reason for choosing ATM for their visit (Reason), practitioner activities (Practitioner Activities), and patient outcomes (Patient Outcome) (Table 4). The Reason was reported in 65 (62.5%) studies. These Reasons were further categorized by accessibility/ease of access (Accessibility), reasons related to COVID-19 (COVID), Study-Based/Referred, and Cost. Accessibility was the reason in 38 (36.5%) of the studies, while COVID, Study-Based/Referred, and Cost were given as reasons in 10, 12, and 5 (9.6%, 11.5%, and 4.8%) studies, respectively.

**Table 4.** Results of Care

	Reported reason	Pre-COVID 19 onset (%, ***n*** = 63)	Post-COVID 19 onset (%, ***n*** = 41)	Total (%, ***n*** = 104)
Reason for patient visit	Accessibility/ease of access	23 (36.5)	15 (36.5)	38 (36.5)
	COVID-19 related	0 (0)	10 (24.4)	10 (9.6)
	Study-based/referred	11 (17.5)	1 (2.4)	12 (11.5)
	Cost	4 (6.3)	1 (2.4)	5 (4.8)
	Total	38 (60.3)	27 (65.9)	65 (62.5)
Practitioner activities	Diagnosis	4 (6.3)	3 (7.3)	7 (6.7)
	Treatment plan	19 (30.2)	19 (46.3)	38 (36.5)
	Referral/follow-up	6 (9.5)	0 (0)	6 (5.8)
	Other	11 (17.5)	8 (19.5)	19 (18.3)
	Total	40 (63.5)	30 (73.2)	70 (67.3)
Patient outcomes	Resolution	16 (25.4)	9 (22.0)	25 (24.0)
	Further care	7 (11.1)	11 (26.8)	18 (17.3)
	Patient experience	5 (7.9)	2 (4.9)	7 (6.7)
	Total	28 (44.4)	22 (53.7)	50 (48.1)

Practitioner Activities were reported in 70 studies (67.3%), which was further divided into four subcategories: Diagnosis, Treatment Plan, Referral/Follow-Up, and Other. Diagnosis, which we defined as those studies that focused on the diagnosis of the patients' condition as the primary activity of the provider during the asynchronous encounter(s), comprised 7 (6.7%) of the studies. The Treatment Plan, which refers to studies in which the primary activity of the provider during the asynchronous encounter(s) was to devise and deliver a treatment plan, was the most common (*n* = 38, 36.5%) of these three subcategories. Referral/Follow-Up describes those studies where the primary activity of the provider as dealing with the handling of further care through referral or additional visits and was included in 6 (5.8%) of the studies.

Examples of Activities in the Other category included giving login information to a patient portal for post-visit access, providing email responses/feedback, and providing primary care physician with information regarding the interaction with the patient.

Fifty (47%) studies reported Patient Outcomes, which were thematically coded into three subcategories: Resolution, Further Care, and Patient Experience. Resolution (*n* = 25, 24.0%) describes studies in which the primary reported patient outcome was a resolution of patient concerns resulting from participating in the encounter. Further Care (*n* = 18, 17.3%) describes studies where the primary patient outcome reported by the study involved the patient concern needing additional steps to reach a point of resolution such as in a follow-up encounter. Patient Experience (*n* = 7, 6.7%) describes those studies where the primary patient outcome was related to patients' perception of the care encounter such as patient satisfaction.

#### Results of care—comparing the literature pre- and post-COVID-19 onset

There do not appear to be large proportional differences between the number of studies reporting on the primary categories related to results of care (Reason, Practitioner Activities, and Patient Outcomes) before and after the onset of COVID-19. However, there were several subcategories with seemingly large proportional differences in the number of studies published relative to COVID-19 onset. Regarding patients' Reasons, it is not surprising that all 10 of 41 (24.4%) studies reporting COVID-19 related reasons for patient use of ATM were published after the onset of the pandemic. Study-based reasons were given more commonly before the onset of COVID-19 (*n* = 11/63, 17.5% pre-COVID-19 onset vs. *n* = 1/41, 2.4% post-COVID-19 onset).

In terms of Practitioner Activities, reporting of treatment plans increased from pre- (*n* = 19/63, 30.2%) to post-COVID-19 onset (*n* = 19/41, 46.34%), while all studies emphasizing Referral/Follow-Up (*n* = 6/63, 9.5%) were published before 2020. In the Patient Outcomes category, the proportion of studies reporting patients needing further care as the primary outcome increased from pre- (*n* = 7/63, 11.1%) to post-COVID-19 onset (*n* = 11/41, 26.8%). All other subcategories from the three major categories appear proportionally similar when comparing the before and after periods of the onset of the COVID-19 pandemic.

### Quadruple aim of medicine

**FIG. 2.**
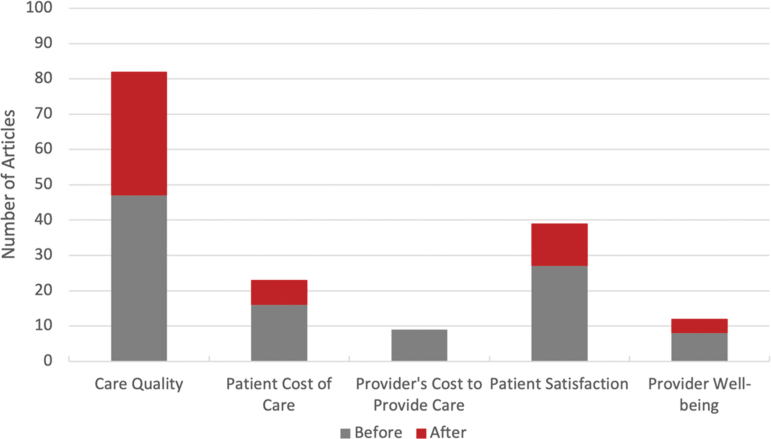
Quadruple aim of medicine.

Consistent with one of our objectives, to ascertain included studies' focus on the aspects of the Quadruple Aim of Health care,^16–19^ we attempted to extract data which addressed different aspects of those four aims from each study (Fig. 2). While we were able to extract data for each category, due to the variability of the data points within each category, no parsimonious system of aggregation was feasible. As such, we recorded whether each aspect of the Quadruple Aim was addressed within each study (i.e., a binary yes/no answer for each aspect for each study). However, because cost affects both patients and providers, but does so differently, we parsed patients' cost of care from providers' cost of providing care, thus creating two categories for the cost aspect of the Quadruple Aim.

Care Quality was the most frequently addressed aspect of the Quadruple Aim of Health care, more than double any other category (*n* = 82,78.8%). Patient Satisfaction was mentioned second most frequently (*n* = 39, 37.5%), followed by patient cost (*n* = 23, 22.1%). Provider Well-being (*n* = 12, 11.5%) and Provider's Cost to Provide Care (*n* = 9, 8.7%) were both infrequently mentioned. All studies addressing Provider Cost of Care were conducted before 2020, while studies addressing Provider Well-being seemed more balanced between the pre- and post-2020 periods.

### Methodological rigor

Ninety-five studies included in this review had a quantitative component. Overall rigor, as measured by the EPHPP,^31,32^ was generally not high. Only 2 studies ranked “Strong” (*n* = 2/95, 2.1%), 4 ranked “Moderate” (*n* = 4/95, 4.2%), and 89 ranked “Weak” (*n* = 89/95, 93.7%; Table 5).

**Table 5.** Methodological Rigor of Included Studies with a Quantitative Component

	Rating	EPHPP category						Global ratings	Modified global ratings	
		Selection bias	Study design	Confounders	Blinding	Data collection methods	Withdrawals and drop-outs	Global rating (%)	Modified EPHPP	Change
Pre-	Strong	3	23	21	2	29	19	2 (3.4%)	6	+4
	Moderate	27	8	0	11	4	4	4 (6.9%)	14	+10
	Weak	28	27	37	45	25	17	52 (89.7%)	38	-14
	N/A						18			
	Total	58	58	58	58	58	58	58 (100.0%)	58	
Post-	Strong	3	5	18	0	7	10	0 (0.0%)	0	0
	Moderate	17	11	0	5	7	4	0 (0.0%)	11	+11
	Weak	17	21	19	32	23	8	37 (100.0%)	26	-11
	N/A						15			
	Total	37	37	37	37	37	37	37 (100.0%)	37	
Overall	Strong	6	28	39	2	36	29	2 (2.1%)	6	+4
	Moderate	44	19	0	16	11	8	4 (4.2%)	25	+21
	Weak	45	48	56	77	48	25	89 (93.7%)	64	-25
	N/A						33			
	Total	95	95	95	95	95	95	95 (100.0%)	95	

EPHPP, Effective Public Health Practice Project; N/A, not applicable.

For qualitative studies and the qualitative components of mixed-method studies (*n* = 17), we used the CASP qualitative evaluation tool.^138^ In Section A, addressing validity of results, all 17 studies had a clear research question but only 10 accounted for all study participants in their conclusions. For Section B, which addresses study design reporting of results, no question was answered in the affirmative more than 12 times. For Section C, only one study was unclear on the applicability of the results (Tables 5 and 6).

**Table 6.** Methodological Rigor of Included Studies with a Qualitative Component

	***Section A***							***Section B***				***Section C***		***Overall***
	***Q1***	***Q2***	***Q3***	***Q4***	***Q5***	***Q6***	***Subtotal (%)***	***Q7***	***Q8***	***Q9***	***Subtotal (%)***	***Q10***	***Subtotal (%)***	***Total (%)***
Yes	17	17	14	17	16	10	91 (89.2)	12	10	11	33 (64.7)	16	16 (94.1)	140 (82.4)
Can't tell	0	0	1	0	0	6	7 (6.9)	0	3	4	7 (13.7)	1	1 (5.9)	15 (8.8)
No	0	0	2	0	1	1	4 (3.9)	5	4	2	11 (21.6)	0	0 (0.0)	15 (8.8)
Total	17	17	17	17	17	17	102 (100.0)	17	17	17	51 (100.0)	17	17 (100.0)	170 (100.0)

## Discussion

In general, the delivery of health care services via telemedicine, and ATM specifically, has increased significantly over the past several decades, but especially since early 2020, due in large part to the inception of the COVID pandemic.^93,139–142^ Overall, we found a dramatic increase in the number of published studies regarding ATM, from 12 published between 2000 and 2010, to 53 published during 2010–2019, and 41 published during 2020–early 2022, mirroring the substantial increase in ATM usage since 2020.

### Study characteristics

Within our included studies, patient characteristics (age, race, gender, rurality, and financial characteristics) were generally not well-reported (e.g., 24% did not report age and 70% did not report race) and, when reported, inconsistencies made connections and interpretations difficult. Among those that reported participant characteristics, participants were more often adults and white, although there were no clear trends regarding gender, rural vs. urban setting, or financial status/type of insurance. However, telemedicine usage varies by multiple factors, including policy, availability, ease of use, and patient characteristics,^139,143,144^ thus limiting our ability to draw meaningful conclusions.

### Alternative terms

We found that the most used term for ATM between patient and provider was “store-and-forward,” followed by “e-visit” and related variants of e-visit. A challenge with using “store-and-forward” is that it is also used to describe communication between physicians using asynchronous telemedical means, leading to confusion on the meaning of the term. It appears that “e-visit” is gaining in usage and popularity, however, which may lead to a de facto resolution of this conundrum.

### Context of care

#### Specialties and credentials

We found a wide variety of health care professionals using ATM, including nurses, nurse practitioners, therapists, dieticians, and dentists, although, unsurprisingly, most providers in included studies were physicians. There was an increase in the number of interprofessional care teams after the inception of the COVID pandemic, which is indicative of a wider variety of provider types using ATM in more recent years. While we were not surprised that dermatology and primary care were the most represented medical specialties, we were surprised that there were not more studies of ATM in psychiatry and mental health.

#### Patient-provider relationship

We found a potential change in the nature of the relationship between provider and patient when using ATM, as the general trends reported in the literature included an increase in ongoing relationships between patient and provider and a decrease in episodic relationships between patient and provider after the onset of COVID-19. This is somewhat intuitive, as ATM allows for patients to still receive care at a distance while also maintaining a more flexible schedule for appointments. It may be too soon to tell if these trends will continue, or if this aberration results from the COVID-19 pandemic.

However, the fact that the use of ATM appears to be growing in ongoing provider-patient relationships suggests that ATM is becoming ever further integrated into the care process and that, as opposed to replacing synchronous care, it is augmenting it, thereby facilitating a smoother overall interaction between provider and patient.^135,145,146^ For example, ATM provides an efficient mechanism for providing follow-up care or feedback after a visit, while also maximizing patient convenience.

### Results of care

#### Reasons for patient use

Previous studies have indicated that overall, reduced travel time and convenience,^147^ in addition to gender and geography,^148^ are among the most frequent reasons for patients choosing telemedicine. This was partially supported in our literature review. Specifically, most patients in these studies reported using ATM technology as their care method due to the much greater ease of access, lower costs, and/or being referred by providers. Although telemedicine has the inherent benefit of not needing patients to make in-person visits to see physicians, the distinct advantage of ATM is the lack of the need for both the patient and provider to be online simultaneously, which offers greater flexibility when scheduling appointments and, thus, makes it a more appealing option to patients.

Examining the reasons for patient use helps identify and affirm certain benefits that ATM has when compared to traditional FTF care and whether ATM is a suitable alternative to FTF care. For instance, the most reported reason for ATM use being accessibility or ease of access confirms that the listed benefits of ATM mentioned earlier (flexible appointment schedules and no need for FTF with provider) are genuine reasons for patients to use ATM over FTF care. Not surprisingly, COVID-19-related reasons were mentioned as the primary reason patients used ATM in 10 articles. The fact that 10 articles mentioned COVID-19 as primary driver of patient use of ATM highlights the impact of the pandemic on patient priorities, such as access and convenience.

While cost-related reasons were not reported as the primary reason as frequently others, their presence reinforces cost as an important consideration in providing care and appears to remain a desirable feature of ATM. Reasons for use may also be co-occurring, such as ease of use and concerns with the pandemic or cost. However, because our search focused on the primary reason given, we cannot provide an analysis of co-occurring reasons for patient use.

#### Practitioner activities

ATM, as opposed to synchronous telemedical services, offers the distinct advantage of not requiring the patient and provider to be present at the same time. Circumstances related to the onset of the COVID-19 pandemic likely accelerated the use of ATM, along with all other forms of telemedicine, during that time. As treatment plans represent a larger proportion of provider activities after the onset of the pandemic, it may be that the use of the technology is becoming more acceptable among providers, rather than a necessity or a subject of study and experimentation.

#### Patient outcomes

Almost half of the included studies reported patient outcomes, which included the Resolution, Further Care, and Patient Experience subcategories. While Resolution was the most frequently reported patient outcome overall, a growing number and proportion of studies published after the onset of COVID-19 reported Further Care as the primary patient outcome, likely necessitated by COVID-19 restrictions. However, this is also consistent with research indicating that, when appropriately integrated, telemedicine serves as an effective and convenient component of the ongoing care experience,^89,149–152^ although patient-, system-, and condition-specific needs should always be taken into consideration when designing integrated telemedical or similar mHealth or eHealth solutions.^89,153,154^ Overall though, there is growing recognition that effective continuity of care will increasingly require the integration of telemedical and traditional care.^150,155,156^

#### Results of care—comparing the literature pre- and post-COVID-19 onset

We observed several pre- to post-COVID-19 onset differences in Practitioner Activities and in Patient Outcomes. Of note is a drop in studies emphasizing practitioner Referral/Follow-Up before 2020 (*n* = 6/63, 9.5% vs. *n* = 0) and an increase in studies reporting patients needing further care as the primary outcome after COVID-19 onset (*n* = 7/63, 11.1% pre-COVID onset vs. *n* = 11/41, 26.8% post-COVID-19 onset). While it may be somewhat semantic, it could signify a maturing body of research and care via asynchronous means, consistent with moving from a practitioner-initiated activity to a standard part of the health care process. This is also consistent with other studies indicating the value of ATM as an augmentation to synchronous care rather than a replacement for synchronous care.^135,145^

### Quadruple aim of medicine

Examining research on ATM through the lens of the Quadruple Aim of Medicine enables one to compare this modality regarding whether it can deliver health care at, or above, the level of traditional FTF care. For example, ATM has clear benefits in terms of its increased accessibility.

In our analyses, we divided the cost component of Quadruple Aim of Medicine into two subcomponents, one focused on Patient Cost of Care and one focused on Providers' Costs to Provide Care. The coverage of the different components of the Quadruple Aim of Medicine in the ATM literature is dominated by examinations of Quality of Care and is most lacking in considerations of both Provider Well-being and Provider Cost to Provide Care, followed by Patient Cost of Care (*n* = 9, 12, and 23 respectively, Table 3). The lack of studies examining the cost of care related to ATM is consistent with studies examining the Quadruple Aim of Medicine in other aspects of telemedicine.^19,145^ We anticipate future gains in these lacking areas as the prevalence of the use of ATM, and research on this topic, increase.

### Methodological rigor

The methodological rigor of quantitative studies included in this review was generally poor, as only 2 (2.1%) studies were rated “Strong” and 4 (4.2%) studies were rated “Moderate,” all of which were published before 2020. However, the lack of studies rated as strong or moderate may, at least in part, be due to the sensitivity of the EPHPP tool to the number of “Weak” ratings across the six categories. For example, the Blinding category contributed more to the overall lack of rigor than all other categories, with 77 (81.1%) “Weak” ratings across all quantitative studies. Confounders was the second most common category for weak ratings across all studies but was only fourth most common in those published since 2020.

With just over half of the included quantitative studies having a retrospective study design, these studies may not have had the benefit of controlling certain sampling and blinding considerations. However, even in prospective telemedicine studies, rigorous study design can be difficult. For example, blinding can be difficult due to requirements related to appropriate medical practice (i.e., ethical considerations). Therefore, we considered a thought experiment wherein the criteria used by the EPHPP was relaxed by one “Weak” rating. The EPHPP typically considers a study “Strong” when no category of the six has a “Weak” rating, “Moderate” when only one has a “Weak” rating, and “Weak” when two or more have “Weak” ratings. By relaxing each of these by one (“Strong” = 0–1 “Weak” ratings, etc.), a slightly more encouraging pattern of overall scores emerges resulting in 6 “Strong” studies, 25 “Moderate” studies, and 64 “Weak” studies.

Studies with qualitative components, compared with the quantitative studies, seemed more rounded and complete, although this may be an artifact of the evaluation tool itself. As we completed the CASP qualitative checklist for each of the applicable studies, we noted difficulties in accounting for all study participants (Q6, part of Section A) and across Section B—indicating difficulties with study design and/or articulating results. Despite some struggles in this category, the qualitative rigor was generally acceptable. However, the CASP Qualitative Checklist does not enable (or the authors' do not recommend) a cumulative “score” for a given study, thus making any judgments based on an overall idea about the quality of qualitative studies difficult. Furthermore, by their nature, qualitative studies tend to be more interpretive, thus making the concise articulation of results more challenging.

### Limitations

We found many inconsistencies in how patient characteristics (e.g., patient race, rurality, financial characteristics) were reported, if at all, which limited the summations that we could provide. While this is not necessarily specific to this area of research, more stringent expectations regarding the reporting patient characteristics would facilitate standardization in reporting and thereby enable the existing knowledge base on this topic to be more robust. With the increasingly apparent need for telemedicine in a global society, medical research should be able to compile compatible data from telemedicine studies, both synchronous and asynchronous.

Also, during the data extraction process, we examined the published date for each article and divided the article into two subsets, those published before 2020 (roughly the pre-COVID period) and those published after 2020. Our findings support the significant increase in research regarding the delivery of health care services via telemedicine over the past several decades, especially since the inception of the COVID-19 pandemic; however, the efficacy of telemedicine, specifically ATM, continually needs to be evaluated and studied in more depth.^157^

Another hurdle we encountered came from the difficulty of simultaneously assessing and summarizing the methodological rigor of a body of research which included quantitative, qualitative, and mixed methodologies. While it is difficult to measure, and subsequently summarize, the rigor across different methodologies, we determined that making an attempt at this summary better suited our three primary goals and provided a better overall view of the research related to ATM.

### Future directions

We believe the examination of coexisting reasons for patients' use of ATM, as well as how patients prioritize those reasons, would be meaningful. Specifically, additional insights into patient behaviors and motivations vis-à-vis ATM would help tailor ATM solutions to patients' needs and preferences. For examples, there is evidence that factors such as demographic characteristics, clinic type, and digital literacy are associated with patient engagement in their own health care—including using similar systems.^158,159^ Also, recent work developing models of digital patient engagement indicates that patient preferences and beliefs are important factors to consider as motivators and de-motivators of patient engagement in their own health care.^160–162^ This, in turn, can affect patient and community health.^163,164^

A possible limitation of ATM as a mode of care delivery is related to its potential vulnerability to fraud. Recent research refutes claims of fraudulent billing behavior, finding that telemedicine-related (including ATM) billing mistakes are rare and are usually due to providers' lack of understanding of payer policies regarding reimbursement.^165^ While this report is encouraging, we note that none of the studies included in this review took fraudulent behavior into consideration. Another area of fraudulent behavior we did not encounter in this review concerns attempts to game the system—such as drug-seeking behaviors. Any time a drug is sought, an in-person visit should probably be involved. However, given the rapid increase in usage of synchronous telemedicine and ATM during the COVID-19 pandemic and the anticipated continued increases, this is an area of telemedicine that we believe should be taken into consideration in future research.

Cost of care to patients is an important consideration in telemedicine and is tied closely to policy. Our findings indicate that Patient Cost of Care via ATM is being addressed, although not as robustly as is needed given the complexity of health care reimbursement systems. In addition, assessing the costs associated with providing care via a new modality can be challenging. Consistent with recent research on e-consultations,^19^ very few studies in this review examined cost to providers or cost effectiveness of providing care in providing care via ATM.

While synchronous telemedicine and FTF care include similar experiences and require similar clinician competencies, ATM is distinct in that it requires different provider competencies.^18,166^ Deep integration of ATM into standard care will require intentional incorporation of training to develop these competencies and the necessary support to successfully carry out asynchronous care in practice. While growing, little standardized telemedical curricular content exists,^167^ and even fewer specifically addressing ATM.

## Conclusion

In this article, we sought to summarize the published peer-reviewed literature related to ATM. We also compared research on ATM before and after the onset of the COVID-19 pandemic and reported notable differences between those time periods. The most used term used to describe ATM between patient and provider was “store-and-forward.” The next most used term was some variant of “e-visit.” However, it appears that “e-visit” is growing in usage while “store-and-forward” is diminishing in usage. In terms of the Quadruple Aim of Medicine in ATM research, Quality of Care is the most researched aspect, while Provider Well-being is the least researched aspect. However, we anticipate more balance as the field grows. Finally, the methodological rigor of studies is generally low—particularly among quantitative studies.

Future ATM research should focus on improving research in terms of Selection Bias and Blinding in quantitative studies, and accounting for study participants and articulating both study design and results in qualitative studies.

## Abbreviations Used

ATM: asynchronous telemedicine
CASP: Critical Appraisal Skills Programme
EPHPP: Effective Public Health Practice Project
FTF: face-to-face
HCV: hepatitis C virus
N/A: not applicable
PRISMA: Preferred Reporting Items for Systematic Reviews and Meta-Analyses
RPM: remote patient monitoring
VDOT: video directly observed therapy
